# 191. Oral Antibiotic Step-Down Therapy for Non-Staphylococcal Gram-Positive Bloodstream Infections

**DOI:** 10.1093/ofid/ofab466.393

**Published:** 2021-12-04

**Authors:** Kelvin Gandhi, Magdalena Wrzesinski, Kristen Bunnell, Ashley Long, Vanessa Hutzley, Rachel Hamilton, John Sundsbak, Allison Gibble

**Affiliations:** 1 Froedtert & the Medical College of Wisconsin, Milwaukee, Wisconsin; 2 Concordia University Wisconsin School of Pharmacy, Milwaukee, Wisconsin; 3 University of Wisconsin-Madison School of Pharmacy, Milwaukee, Wisconsin

## Abstract

**Background:**

Bloodstream infections are traditionally treated with intravenous (IV) antimicrobial therapy, which may increase length of stay and healthcare costs. The purpose of this study is to evaluate if oral antibiotic step-down therapy for non-staphylococcal gram-positive bloodstream infections (GP-BSIs) is non-inferior to IV antibiotics.

**Methods:**

This single-center, retrospective cohort study included patients with a non-*Staphylococcus aureus*, non-*Staphylococcus lugdunensis* GP-BSI from January 2017 to December 2019. Patients were excluded if they fit any of the following criteria: organism identified as contaminant, polymicrobial BSI, recurrent BSI within the past 90 days, or receipt of an effective antibiotic for a duration longer than what is indicated for BSI treatment. Patients were categorized into those who received an IV antibiotic for the total duration of therapy and those who received an oral step-down antibiotic for at least one-third of the treatment course. The primary composite outcome was the incidence of 90-day clinical failure consisting of 90-day all-cause mortality, change in therapy due to inadequate clinical response, and 90-day BSI recurrence. The secondary outcomes included the individual components of the primary composite outcome, line-related complications, and hospital length of stay. Bivariate analysis was conducted to assess for predictors of 90-day clinical failure.

**Results:**

A total of 308 patients were included (oral group, n=94; IV group, n=214). Pitt Bacteremia Scores were low overall, but higher in the IV group (0 vs 1, *p*=0.045). The oral group had a higher proportion of GP-BSI caused by streptococcal species (76% vs 61%, *p*< 0.001). The oral group had a lower incidence of 90-day clinical failure and was found to be noninferior to the IV group (9% vs 14%; mean difference -5%, 90% CI -12.7 to 2.6). The IV group had a longer hospital length of stay (4 vs 6 days, *p*< 0.001), however there were no other significant differences in secondary outcomes. Bivariate analysis found no significant predictors of 90-day clinical failure.

**Conclusion:**

Oral antibiotic step-down therapy was found to be non-inferior to IV antibiotic therapy, and thus may be an alternative option for the treatment of non-staphylococcal GP-BSIs.

**Disclosures:**

**All Authors**: No reported disclosures

